# Transcranial Doppler in the Detection of Cerebral Vasospasm After Subarachnoid Hemorrhage

**DOI:** 10.7759/cureus.61569

**Published:** 2024-06-03

**Authors:** Maliha Hakim, Ghulam Kawnayn, Mohammad Sayeed Hassan, Mohammad Nur Uddin, Mashfiqul Hasan, Muhammad Rezeul Huq

**Affiliations:** 1 Neurology, National Institute of Neurosciences and Hospital, Dhaka, BGD; 2 Neurology, Combined Military Hospital, Dhaka, BGD; 3 Endocrinology, Diabetes, and Metabolism, National Institute of Neurosciences and Hospital, Dhaka, BGD

**Keywords:** sah complications, transcranial doppler, subarachnoid hemorrhage, complications of sah, delayed cerebral ischemia (dci), cerebral vasospasm

## Abstract

Background

Transcranial Doppler (TCD) is a simple, noninvasive, nonionizing, portable technique but not widely practiced to detect cerebral vasospasm after subarachnoid hemorrhage (SAH).

Objective

The aim of this study was to assess the performance of TCD in the detection of cerebral vasospasm in patients with SAH considering CT angiography (CTA) as a gold standard.

Methods and material

This cross-sectional study included 50 patients with acute SAH admitted to the National Institute of Neurosciences & Hospital (NINS & H), Dhaka, Bangladesh, from February to June 2021. The neurological status, severity of SAH, and initial CT findings were recorded. All patients were screened for cerebral vasospasm with TCD on the 4th, 7th, 10th, and 14th days after the event. Screening of cerebral vasospasm by CTA was done on the 14th day of the event or earlier if TCD suggested vasospasm.

Results

The mean age of the participants was 51.4 ±13.4 years (mean ± SD), and females were predominant (N=29, 58%). CTA detected cerebral vasospasm in 18 (36%) participants, but TCD could detect it in only 13 (26%) cases. Among the participants who had no vasospasm by CTA, all but one were also found to have no vasospasm by TCD. The agreement between TCD and CTA in detecting cerebral vasospasm was significant (p<0.001, κ=0.726). TCD shows good specificity (96.9%) and positive predictive value (92.8%), but sensitivity (72.2%) and negative predictive value (81.6%) were comparatively lower. Overall, the diagnostic accuracy of TCD in detecting cerebral vasospasm was 88%.

Conclusions

Although compared to CTA, TCD is a highly specific but less sensitive tool in detecting vasospasm, TCD remains a reliable screening tool for detecting vasospasm following SAH.

## Introduction

Cerebral vasospasm indicates sustained contraction of the cerebral arteries that can occur after subarachnoid hemorrhage (SAH) and may lead to delayed cerebral ischemia (DCI) [1]. The vasospasm after SAH usually starts on days 3-7, typically increasing on the fourth day after SAH and gradually decreasing following the 14th day [2]. DCI or cerebral vasospasm can be detected by clinical features and different modalities of vascular imaging [3]. Severe vasospasm may cause greater vessel narrowing with a reduction of blood flow to ischemic thresholds and the development of DCI [4]. Detection of severe vasospasm and timely intervention to prevent DCI may prevent morbidity and mortality imposed by SAH. Hence, much interest has been focused on developing effective preventive, diagnostic, and therapeutic measures for vasospasm [5].

The optimal screening modality for detecting symptomatic cerebral vasospasm is yet to be determined. The gold standard is digital subtraction angiography (DSA), but this is an invasive procedure that carries a substantial complication risk [6]. Transcranial Doppler (TCD) and/or computed tomography angiography (CTA) are commonly used as noninvasive screening tools [7]. DSA can detect cerebral vasospasm in up to 70% of SAH patients, although only 30% of them are clinically symptomatic [3,7]. CTA also has good sensitivity and specificity in detecting cerebral vasospasm. However, it has radiation and contrast-induced hazards. It is costlier than TCD, and not suitable for critically ill patients to perform CTA frequently. TCD is accepted as a simple, noninvasive, nonionizing, portable technique that indirectly estimates cerebral blood flow (CBF) by measuring the CBF velocities. TCD, a noninvasive tool, allows for bedside monitoring and can detect cerebral vasospasm even in clinically asymptomatic patients [8,9]. Since velocities in a given artery are inversely proportional to the respective cross-sectional area, serial TCD evaluations can detect evolving vasospasms in patients following SAH [10,11].

 SAH is among the top five causes of mortality in the Neurology Department [12]. Mortality and morbidity after hospital admission often depend on the development of DCI. It is necessary to detect vasospasm at the earliest to take measures for the prevention of DCI. Although very effective in detecting vasospasm, angiogram procedures are not available bedside, are invasive and costly, and carry radiation hazards. TCD can be an attractive alternative for the detection of cerebral vasospasm in these groups of patients. However, TCD is not widely available in Bangladesh. Moreover, being operator dependent it has some limitations. Before advocating its widespread use, TCD needs to be validated in our setting against a well-established angiogram procedure to detect vasospasm. A recent meta-analysis showed that TCD may be very useful in identifying patients with vasospasm (high positive predictive value) but has limited capacity to rule out it (low negative predictive value) [13]. In this background, the current study aims to compare the agreement of a relatively recent but easily applicable bedside procedure TCD with well-established CTA in the detection of cerebral vasospasm in our cohort with SAH.

## Materials and methods

Study design and population

This was a cross-sectional study carried out from February to June 2021 in the Department of Neurology, NINS & H. A total of 50 adult patients with SAH admitted to the Neurology Department within the first 48 hours of the ictus were enrolled by purposive sampling. Critically ill patients with terminal illness or multi-organ failure and patients with renal impairment or contraindications for contrast agents were excluded.

Study procedure

The patient’s neurological status was recorded and then the severity of the neurological deficit was evaluated using the Hunt and Hess scale. The severity of SAH was assessed using a modified Fissure scale based on computed tomography (CT) imaging. Aneurysmal bleeding was confirmed by CTA.

TCD protocol

Following standard protocol, all patients were screened for cerebral vasospasm with a TCD device (Atys-Médical, Soucieu en Jarrest, France) on the 4th, 7th, 10th, and 14th day after the event. The following vessels were examined by TCD: extracranial internal carotid artery (ICA), middle cerebral artery (MCA), anterior cerebral artery (ACA), basilar artery (BA), and posterior cerebral artery (PCA). Vasospasm in the MCA was defined as Lindegaard ratio (LR)>3 and mean flow velocity (MFV)>120 cm/sec [7,14-16]. The LR was calculated as MCA-MFV divided by the distal ipsilateral extracranial ICA-MFV [14,15]. Severe vasospasm in the MCA was defined by an MFV>200 cm/sec and LR>6 [7,14,16]. Vasospasm of the PCA and BA was defined as MFV>85 cm/sec [7,16]. MFV>80 cm/sec with a Sloan ratio of >4.0 was considered a vasospasm for the ACA [7,16]. The Sloan ratio was calculated as ACA-MFV divided by ipsilateral ICA-MFV [7,16]. Any vasospasm on TCD in the PCA, BA, and ACA was regarded as severe because TCD cannot differentiate in severity in these vessels [7]. The most severely affected vessel on TCD and CTA was used for analysis.

CTA protocol

Screening of cerebral vasospasm by CTA was done on the 14th day of the event or earlier if TCD suggests vasospasm. On CTA, vasospasm was defined as luminal reduction relative to the proximal and distal segments, in comparison to the contralateral vessels. Initial CTA was performed within three days of the event to identify the aneurysm and exclude atherosclerotic narrowing and hypoplasia as mimics of cerebral vasospasm. If technical causes such as coil or clip artifacts lead to the evaluation of vessels being impossible, that was noted as not assessable.

Data analysis

All data were recorded in a preformed data collection sheet. Analyses were performed with SPSS software, Version 22.0 (IBM Corp., Armonk, NY). Normally distributed continuous data were expressed in terms of mean and standard deviation. Skewed continuous data were presented in terms of median and interquartile range (IQR). Categorical or discrete data were summarized in terms of frequency and percentages. The Kappa test was used to see the agreement between TCD and CTA. A two-sided p-value of less than 0.05 was considered to indicate statistical significance.

Ethical consideration

Ethical clearance for the study was taken from the Institutional Review Board of NINS & H (IRB/NINS/2023/325). All study subjects or their next of kin were informed about the nature, purpose, and implications of the study, as well as the entire spectrum of benefits and risks of the study. Strict confidentiality was maintained in dealing with study subjects. Informed written consent of all the study subjects was taken.

## Results

This study involved 50 patients with SAH admitted within 48 hours of an acute event. Table 1 shows the characteristics of the study participants. The mean age of the participants was 51.4 years (SD 13.4 years), and females were predominant (N=29, 58%). The median clinical severity defined by Hunt and Hess grade was 3 (IQR 2-3). Of the participants, 11 (22%) had hypertension and five (10%) had diabetes. The anterior communicating artery was the most common location of a cerebral aneurysm (N=19, 38%) followed by ICA (N=13, 26%), MCA (N=7, 14%), and BA (N=2, 4%). In 18% of the participants, no aneurysm could be detected by initial CTA.

**Table 1:** Characteristics of the study participants SD, standard deviation; IQR, interquartile range; ACOM, anterior communicating artery; MCA, middle cerebral artery; ICA, internal carotid artery

Characteristics	Value
Age (mean±SD; years)	51.4±13.4
Gender	
Male	21 (42%)
Female	29 (58%)
Hunt and Hess grade (median, IQR)	3 (2-3)
Modified Fisher scale score (median, IQR)	3 (2-3)
History of hypertension	11 (22%)
History of diabetes	5 (10%)
Aneurysm location	
ACOM	19 (38%)
MCA	7 (14%)
ICA	13 (26%)
Basilar	2 (4%)
Not-detected	9 (18%)

CTA detected cerebral vasospasm in 18 (36%) participants, but TCD could detect it in only 13 (26%) cases. Among the participants who had no vasospasm by CTA, all but one were also found to have no vasospasm by TCD. The agreement between TCD and CTA in detecting cerebral vasospasm was substantial and significant (p<0.001, κ=0.726) (Table 2).

**Table 2:** Agreement between TCD and CTA in detecting cerebral vasospasm By Kappa test p<0.001, κ=0.726 CTA, computed tomography angiography; TCD, transcranial Doppler

Vasospasm in TCD	Vasospasm in CTA		
	Present	Absent	Total
Present	13 (72.2%)	1 (3.1%)	14 (28.0%)
Absent	5 (27.8%)	31 (96.9%)	36 (72.0%)
Total	18 (100.0%)	32 (100.0%)	50 (100.0%)

Table 3 shows the performance of TCD in the detection of cerebral vasospasm taking the finding of the CTA as gold standard. TCD shows good specificity (96.9%) and positive predictive value (92.8%), but sensitivity (72.2%) and negative predictive value (81.6%) were comparatively lower. The diagnostic accuracy of TCD in detecting cerebral vasospasm was 88%.

**Table 3:** Performance of TCD in the detection of cerebral vasospasm TCD, transcranial Doppler

Parameters	Value	95% CI
Sensitivity	72.2%	46.52-90.31
Specificity	96.9%	83.78-99.92
Positive predictive value	92.8%	64.90-98.92
Negative predictive value	81.6%	74.59-92.90
Accuracy	88.0%	75.69-95.47

## Discussion

SAH is a serious condition that can often lead to mortality among neurology patients. In Bangladesh, our tertiary center serves as the only referral neuroscience institute, and we recently conducted a study that enrolled 50 patients with SAH admitted within 48 hours of the acute event; 58% of our participants were female. Two large studies of SAH with 580 and 415 patients also revealed that females were more affected (68% and 60.5%, respectively) [17,18]. Our findings showed a high proportion of aneurysms in the anterior communicating artery, which is consistent with previous studies [19].

To identify early and evolving vasospasm, we repeatedly used TCD on the fourth to 14th day of the event, as cerebral vasospasm is a dynamic process that starts within 72 hours of the acute event and peaks at around seven days. Our study found a few false-negative cases that had evidence of vasospasm on CTA. However, we hardly observed any false-positive TCD cases (only one out of 50), indicating a high specificity and a relatively lower but fair level of sensitivity. Our results are similar to the recently published meta-analysis [13], which also showed a high specificity of 89.5% (95% CI: 80.3-94.7) and a pooled sensitivity of 66.7% (95% CI: 55.9-75.9) for the middle cerebral artery (10 studies, 1,408 tests). In another prospective cohort study, TCD was compared with CTA in predicting DCI. TCD had a sensitivity of 44% on day 5 and specificity of 67% [7]. On the other hand, a prospective observational study in Vietnam showed 95% sensitivity and 91% specificity, considering CTA as the comparison tool [20]. In another retrospective study [21], TCD was compared with conventional angiography having a sensitivity of 80%-90% (95% CI: 0.77-0.96) but with a corresponding specificity of 50% (95% CI: 0.40-0.56).

The sensitivity and specificity vary from artery to artery, with a maximum for MCA (sensitivity of 67% and specificity of 99%) and BA (sensitivity of 76.9% and specificity of 79%) [21]. Other arteries such as ACA, PCA, and vertebral arteries have a sensitivity of less than 50% [22].

Although TCD has a relatively low sensitivity, it is a noninvasive bedside test with high specificity and can be used for daily monitoring of non-ambulatory patients. Also, TCD has the advantage of having no hazards of radiation or contrast-induced adverse effects [22]. TCD can be performed in low-resource settings and may be the preferred method in remote areas. It can be a game-changer in managing SAH patients in developing countries of Asia and Africa, where CTA is not widely available. Other methods such as CT perfusion [23] and black-blood MR angiography (BBMRA) [24] can detect DCI with high sensitivity and specificity, but they are expensive and not widely available. TCD has some other limitations also. It can be a time-consuming examination with operator dependency. A good bony window is needed to visualize some arteries [25]. The presence of cerebral hemorrhage can be detected in TCD as hyperechoic shadows, which may lead to acoustic limitations in some cases [26]. However, daily TCD monitoring can be challenging due to logistical constraints. It requires a significant amount of the operator's time and may create management problems. Despite these limitations, TCD can be a reliable and cost-effective screening tool for early and bedside detection of vasospasm in patients with SAH.

However, our study has some limitations. Our sample size was moderate, and it was a single-center study, which may limit its generalizability. Additionally, we did not assess the relation of cerebral vasospasm with the outcome or prognosis of the patient. Moreover, we performed TCD on some fixed dates, not daily, which might have missed some DCI cases. Finally, since DSA is an invasive procedure, it was not used in the current study, which may have limited our ability to detect all cases of cerebral vasospasm following SAH. Further research is recommended to assess the usefulness of TCD in SAH patients, using DSA as the comparison tool.

## Conclusions

TCD is highly specific but less sensitive in detecting CTA-proven cerebral vasospasm following SAH. Considering its diagnostic accuracy, this non-invasive tool may be used as a reliable screening investigation to detect cerebral vasospasm following SAH. Moreover, as TCD is a bedside test, it can be the preferred option for critically ill patients. Early detection of cerebral vasospasm will help clinicians to take therapeutic intervention to prevent permanent neurological deficit.
